# Evaluation of the Thermal Stability and Micro-Modification Mechanism of SBR/PP-Modified Asphalt

**DOI:** 10.3390/polym16040456

**Published:** 2024-02-06

**Authors:** Qing Zhang, Dehua Hou, Zhongyu Li, Hainian Wang, Shi Dong

**Affiliations:** 1 Henan Key Laboratory of High-Grade Highway Detection and Maintenance Technology, Xinxiang 453003, China; zhangqing@htu.edu.cn (Q.Z.); keyan01@chngaoyuan.com (D.H.); plan@chngaoyuan.com (Z.L.); 2 National Engineering Research Center of Highway Maintenance Equipment, Xinxiang 453003, China; 3 Collaborative Innovation Center of Henan Province for Fine Chemicals Green Manufacturing, School of Chemistry and Chemical Engineering, Henan Normal University, Xinxiang 453007, China; 4 Key Laboratory of Green Chemical Media and Reactions, Ministry of Education, School of Chemistry and Chemical Engineering, Henan Normal University, Xinxiang 453007, China; 5 School of Highway, Chang’an University, Xi’an 710064, China; wanghn@chd.edu.cn; 6 College of Transportation Engineering, Chang’an University, Xi’an 710064, China

**Keywords:** SBR/PP particles, modified asphalt, rheological behavior, Cole–Cole diagram, fluorescence microscope

## Abstract

To evaluate the thermal stability of composite polymer-modified asphalt, thermoplastic elastomer styrene-butadiene rubber (SBR)/polypropylene (PP) pellets were prepared using a hot-melt blending technique, with butyl rubber powder and waste polypropylene pellets as raw materials. The effects of different evaluation indexes on the thermal stability of SBR/PP-modified asphalt were investigated using a frequency scan test and a multi-stress creep recovery (MSCR) test, and the compatibility of SBR/PP particles with asphalt was studied using the Cole–Cole diagram and microstructure images. The tests show that, firstly, the performance grade (PG) classification of asphalt can be improved by adding an SBR/PP thermoplastic elastomer to enhance the adaptability of asphalt in high- and low-temperature environments, and the evaluation separation index can reflect the high-temperature storage stability of composite-modified asphalt more reasonably. Additionally, the larger the rubber-to-plastic ratio the worse the high-temperature thermal stability of composite-modified asphalt. Moreover, the addition of additives to the composite particles can promote the SBR/PP particles in the asphalt to be more uniformly dispersed, forming a more desirable microstructure and improving the thermal stability of composite-modified asphalt. Ultimately, the semicircular curve of the Cole–Cole diagram can reflect the compatibility characteristics of the two-phase structure of SBR/PP-modified asphalt, which can be used as an auxiliary index to evaluate the compatibility of polymer-modified asphalt.

## 1. Introduction

The increase in traffic load and volume year after year and the significant impact of seasonal shifts results in frequent damage to asphalt pavement. In order to prevent or reduce this, researchers have gradually paid attention to improving the performance of asphalt by using polymers. By adding polymers, the rheological and mechanical properties of asphalt can be improved, which helps to optimize the viscoelastic behavior of asphalt [1,2]. However, the influence level of different types of polymers on asphalt performance may vary, and it is not easy to improve the comprehensive mechanical properties of asphalt at the same time by using only one polymer. Therefore, it is necessary to use different types of polymers in a mixed manner. For example, polyethylene/polypropylene (PE/PP) [3], styrene-butadiene rubber/styrene-butadiene-styrene (SBR/SBS) [4], SBR/crumb rubber (CR) [5], phenolic resin (PF)/SBR [6] and other composite polymers are used to modify asphalt. This helps it to better play the role of different types of polymers in improving the rheological properties of asphalt, and to give it the dual properties of composite polymers.

PP is widely used in road engineering as a recyclable thermoplastic polymer. This is due to its high strength and resistance to heat and corrosion [7,8]. Duggal et al. [9] applied hard waste plastic PP to a modified asphalt mixture, which not only improves the temperature sensitivity of the asphalt mixture, but also reduces the engineering cost and forms an environmentally friendly, green pavement. Jerin et al. [10] pointed out that adding thermoplastic PP to asphalt can significantly improve the high-temperature deformation resistance of asphalt, but it will reduce the ductility of asphalt. SBR can significantly improve the viscosity, toughness and low-temperature flexibility of asphalt. However, the high-temperature deformation resistance of SBR-modified asphalt was relatively weak [11]. Vamegh et al. [12] used PP and SBR composite particles to modify asphalt. The research showed that the high-temperature deformation resistance and low-temperature crack resistance of the SBR/PP composite-modified asphalt mixture were improved, and its fatigue life was 50% higher than that of SBS-modified asphalt. Wang et al. [13] produced PPA-SBS-modified bio-blend bitumen by adopting polyphosphoric acid (PPA) and styrene-butadiene-styrene (SBS), which has been experimentally proven to possess excellent thermal cracking and fatigue resistance.

However, there is a lack of in-depth research on the stable state of composite-modified asphalt in the process of preparation and application, especially in a high-temperature environment. The performance change range of different polymers is different, and the phase separation of different polymers is significant, resulting in the quality of composite-modified asphalt being difficult to control. Compared with SBR-modified asphalt, the chemical system is more stable, but its stable ageing is still less than 5 h [14]. Ren Z et al. [15] pointed out that the difference in density between polymers and asphalt is the main problem causing concern over the storage stability of phase separation. By improving the compatibility between polymers and asphalt, the storage stability of polymer-modified asphalt can be improved. The modified asphalt can remain macroscopically uniform and stable for a long time. Therefore, the rubber–plastic alloy material is prepared using plasticizing technology. This is because each component of its two-phase structure can fully dissolve into the other and enhance its winding ability. It is difficult to control the relative movement of polymer molecules in the process of asphalt dispersion, which hinders the occurrence of polymer separation, thereby reducing the composite polymer. The density difference in asphalt could be effectively improved by the thermal stability of composite-modified asphalt [16]. Rohayzi et al. [17] reviewed polyphosphoric acid, Evotherm, mangosteen powder, trimethyl-quinoline and sulfur related to the additives that can improve the properties of rubberized asphalt. Liu et al. [18] have found that montmorillonite (Mt) nano clay could act as an effective modifier by adding Mt nano clay to crumb rubber-modified bitumen binder.

This work first used the plasticizing technology of an open mill to mix powder and recycled polypropylene into SBR/PP thermoplastic elastomer. Finally, the compatibility between SBR/PP thermoplastic elastomer and asphalt is studied using the Cole–Cole diagram and microstructure image, so as to understand better the improvement mechanism of SBR/PP thermoplastic elastomer on asphalt, and provide an experimental basis for the application of research into SBR/PP thermoplastic elastomer modified asphalt.

## 2. Materials and Laboratory Test

### 2.1. Materials

Styrene-butadiene rubber powder is SBR 1502, which is produced by the Zhangmutou plastic chemical industry in Dongguan City, Guangdong province, China. Polypropylene is recycled PP particles, produced by Hunan Yinghong New Materials Co., Ltd. in Loudi, Hunan Province, China. The compatibilizer is a self-made maleic anhydride monomer; the crosslinking agent is tetramethyl thiuram monosulfide (TMTM). SK 70 asphalt was selected for the test, and the main performance indicators are shown in Table 1.

### 2.2. Preparation of SBR/PP Composite-Modified Asphalt

**Figure 1:**
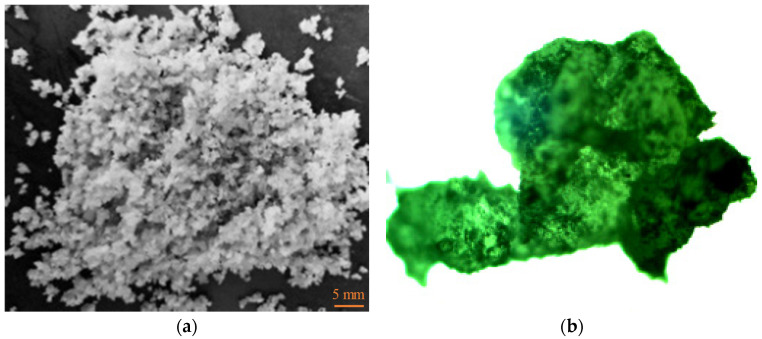
Appearance and particles of SBR/PP thermoplastic elastomer. (**a**) Particles of SBR/PP thermoplastic elastomer. (**b**) General optical micrograph of SBR/PP thermoplastic elastomer.

1. The SBR powder and PP granules were weighed according to the proportioning design, respectively, mixed in a mixer for 1 min, and then added to an appropriate amount of compatibilizer, a crosslinking agent and an auxiliary agent for blending; finally, the blend was added to a screw extruder for extrusion. The temperature of the extruder feeding port was set to 180 °C, the temperature of the die head was set to 190 °C, and the rotational speed of the screw was 150 r/min to obtain the SBR/PP thermoplastic elastomer. In order to improve the dispersion and swelling of SBR/PP thermoplastic elastomer in asphalt, it is ground by a rubber pulverizer [19]. The effect is shown in Figure 1. It can be observed through the general optical micrograph microscope that the surface of the SBR/PP thermoplastic elastomer particles is rough, the shape is irregular, and the SBR and PP are evenly mixed. SBR/PP elastomers are not white, but off-white (shown in Figure 1a). It looks off-white mainly due to the use of auxiliary lighting during the shooting process, making the overall color bright (or off-white). In this study, the average particle size of SBR particles is approximately 20 mesh. According to some relevant studies [20,21,22], PP was calculated and revealed to exhibit isotactic configuration, with a molecular weight of 40,000 g/mol; this constitutes over 95% of the entire polymer content.

2. The SK 70 base asphalt was heated to a hot-melt state, and then the SBR/PP thermoplastic elastomer was slowly added to the asphalt. Then the modified asphalt was heated to 170~180 °C and mechanically stirred for 20 min, so that the SBR/PP thermoplastic elastomer body was fully swollen in asphalt. Then, the modified asphalt was sheared with a high-speed shearer at the speed of 5000 R/min for 40 min [23]. Finally, SBR/PP composite-modified asphalt was obtained. The proportion design of SBR/PP thermoplastic elastomer is shown in Table 2, in which the content of SBR/PP thermoplastic elastomer is 4% of the mass of asphalt.

### 2.3. Analysis and Testing

In the test, the evaluation indexes of penetration, softening point, ductility [24], softening point difference and PG [25] were used to evaluate and analyze different modified asphalts. At the same time, the frequency sweep test was carried out on different modified asphalts. The applied load frequency range was 1~100 rad/s, the test temperature was 60 °C, and the elastic modulus *G*′ and viscous modulus *G*″ of the modified asphalt were measured.

The multiple-stress creep recovery (MSCR) test evaluated the delayed elastic recovery performance of the modified asphalt [26]. The test adopts the intermittent cyclic loading method of “loading-unloading” for the sample by DSR. The stress of 0.1 kPa was first loaded, and the intermittent cyclic loading was performed for 10 cycles; one cycle of 10 s—of which the first 1 s was the loading deformation stage and the last 9 s was the unloading recovery stage—then a stress of 3.2 kPa was loaded and the procedure repeated. The total test time was 200 s, and the test temperature was 60 °C. The recovery percentage *R* and the non-recoverable creep compliance *J_nr_* of the modified asphalt were measured.

In addition, rheological property testing has been proven to be an effective method for evaluating storage stability, so a multi-stress creep evaluation test can be performed on the modified asphalt on the top and bottom of the sample to evaluate the thermal stability of modified asphalt. Separation tests of SBR/PP composite-modified asphalt were evaluated according to JTG-E20-2011 [27]. Tubes with modified asphalt were stored in ovens at 163 °C for 48 h, then frozen at −5 °C for 5 h before being divided into three sections. The softening point difference and separation index of the upper and lower sections were tested [28]. The separation index is defined as follows:

(1) $$separation\;indexR=\frac{\left|R_{top}-R_{bottom}\right|}{R_{ave}}$$

where *R_top_* is the per cent recovery from the top container, and *R_bottom_* is the per cent recovery from the bottom container.

(2) $$separation\;indexJ_{nr}=\frac{\left|J_{nr\text{-}top}-J_{nr\text{-}bottom}\right|}{J_{nr\text{-}ave}}$$

where *J_nr-top_* is the non-recoverable creep compliance from the top container, and *J_nr-bottom_* is the non-recoverable creep compliance from the bottom container.

In order to study the compatibility between SBR/PP thermoplastic elastomer and asphalt and its improvement mechanism, the microstructure of modified asphalt was analyzed using the HDS-BFS-BK6000 fluorescence microscope manufactured by Hydestar Technology (Beijing) Co., Ltd. in Beijing China.

## 3. Results and Discussion

### 3.1. Technical Indicators of SBR/PP Composite-Modified Asphalt

In this study, penetration, softening point, ductility and softening point difference were used to evaluate the mechanical properties of different modified asphalts. The test results are shown in Table 3. Compared with the matrix asphalt, the penetration of the SBR/PP composite-modified asphalt decreased, and the softening point and ductility increased, indicating that its anti-deformation and anti-cracking abilities were improved. With the increase in the rubber-to-plastic ratio, *M_SBR_*:*M_PP_*, the performance of SBR/PP composite-modified asphalt changed. The penetration and ductility of modified asphalt gradually increased, indicating that its ability to resist shear failure decreased, while the ductility at low temperature improved. The decrease in the softening point of modified asphalt indicates that its temperature sensitivity was relatively poor [29]. When the compatibilizer was added, the ductility of the SBR/PP composite-modified asphalt reached the maximum, an increase of 71% compared with sample A (5:5), and its low-temperature crack resistance was outstanding. The addition of a crosslinking agent has no significant effect on its properties.

From the evaluation index of softening point difference, it can be seen that the high-temperature storage stability of SBR/PP-modified asphalt with different ratio designs had a slight change range. The extreme value of test data was 0.4 °C, and the data dispersion coefficient was 0.083. The results show that the softening point difference had a poor ability to distinguish the thermal stability of SBR/PP-modified asphalt.

### 3.2. PG Analysis of SBR/PP Composite-Modified Asphalt

PG is the asphalt performance classification standard in the Strategic Highway Research Program (SHRP) in the United States [30]. The study of PG classification of SBR/PP composite-modified asphalt has a robust guiding role for its practical application. It can be seen from the PG grading test results of different modified asphalts (Table 4) compared with the base asphalt, that the high-temperature PG of SBR/PP composite-modified asphalt can be increased by up to three grades, and its anti-rutting ability is significantly improved. With the increase in the rubber-to-plastic ratio, *M_SBR_*:*M_PP_*, the high-temperature deformation resistance of the modified asphalt decreases, indicating that the high-temperature mechanical properties of polypropylene plastics are better than that of styrene-butadiene rubber [31]. After adding a compatibilizer and crosslinking agent, SBR/PP composite-modified asphalt has the most comprehensive PG range and the best high- and low-temperature rheological properties.

In order to ensure that the bonding material does not produce fatigue cracking, the fatigue factor G*·sin *δ* at medium temperature should not exceed 5 Mpa [32]. Otherwise, it is considered that the bonding material is prone to fatigue cracking failure. It can be seen from Table 4 that, with the increase in the rubber-to-plastic ratio, *M_SBR_*:*M_PP_*, the G*·sin *δ* of the SBR/PP composite-modified asphalt gradually decreases, indicating that the fatigue cracking risk of modified asphalt decreases with the increase in the proportion of styrene-butadiene rubber. The addition of the compatibilizer can increase the G*·sin *δ* of the modified asphalt and weaken the fatigue cracking resistance; when the compatibilizer and crosslinking agent are added, the G*·sin *δ* of the modified asphalt is the smallest, and this modified asphalt has the best resistance to fatigue cracking.

The BBR test is mainly used to evaluate the low-temperature performance of asphalt [33]. From the test results, when *M_SBR_*:*M_PP_* is 3:7, the PG of modified asphalt is the highest under low-temperature conditions, and the revised asphalt becomes relatively hard and brittle, and prone to low-temperature cracking. This shows that SBR/PP thermoplastic elastomer with a low rubber-to-plastic ratio is not conducive to the application of modified asphalt in the low-temperature setting. In contrast, SBR/PP thermoplastic elastomer with higher styrene-butadiene rubber content is conducive to improving the SBR/PP composite modification low-temperature cracking resistance of asphalt. The addition of compatibilizer can further reduce the low-temperature PG classification of SBR/PP composite-modified asphalt, which shows that the low-temperature cracking resistance and temperature sensitivity of SBR/PP composite-modified asphalt is relatively good [34], and that cracking does not easily occur with the sudden drop in ambient temperature.

To sum up, the PG classification of asphalt can be improved by adding SBR/PP thermoplastic elastomer to enhance the adaptability of asphalt in high- and low-temperature environments [35]. When compatibilizers and crosslinkers were added to SBR/PP thermoplastic elastomer, the resistance of SBR/PP composite asphalt to rutting was further improved. The low-temperature performance of the modified asphalt has no significant negative impact, and the comprehensive rheological performance is relatively the best.

### 3.3. Stability Analysis of SBR/PP Composite-Modified Asphalt

**Figure 2:**
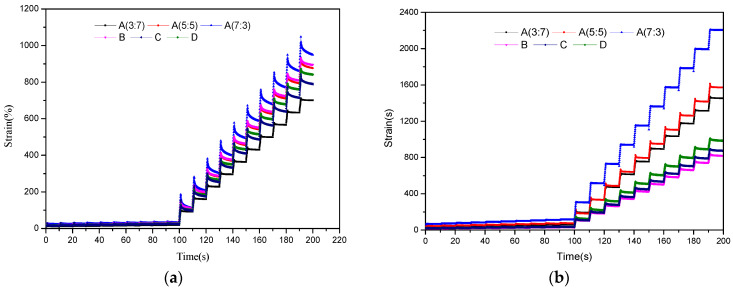
MSCR test loading results of SBR/PP composite-modified asphalt at stress levels of 0.1 and 3.2 kPa. (**a**) Top part of the tube for modified asphalt. (**b**) Bottom part of the tube for modified asphalt.

The creep and deformation recovery characteristics of the SBR/PP composite-modified asphalt were studied based on the MSCR test, so as to evaluate the difference in the rheological properties of the top and bottom of the modified asphalt separation tube. The time–strain curve of the top position of the SBR/PP composite-modified asphalt separation tube is shown in Figure 2a. It can be seen that, with the increase in the rubber-to-plastic ratio, the cumulative strain of modified asphalt gradually increases, and the resistance to deformation gradually weakens. When only the compatibilizer was added to the SBR/PP thermoplastic elastomer, the strain change amplitude of the modified asphalt on the top of sample B was not obvious relative to sample A (5:5); when the compatibilizer and the crosslinking agent were used, the strain amount of modified asphalt on the top of sample D was slightly lower than that of sample A (5:5).

The time–strain curve of the sample at the bottom of the SBR/PP composite-modified asphalt separation tube is shown in Figure 2b. It displayed that the strain variation amplitude of the modified asphalt at the bottom of sample A (7:3) was significantly larger than that of other samples. When a compatibilizer or a crosslinking agent was used, the strain of the modified asphalt in the bottom position of the separation tube was relatively reduced.

To sum up, the time–strain curves of the SBR/PP-modified asphalt at the top and bottom of the separation tube are obviously different, which is mainly due to the difference in the compatibility between asphalt and SBR/PP thermoplastic elastomer with different proportion design, resulting in extra thermal stability of SBR/PP composite-modified asphalt [36]. Therefore, it is necessary to further analyze its performance through the recovery percentage *R* and the unrecoverable creep compliance *J_nr_*.

**Figure 3:**
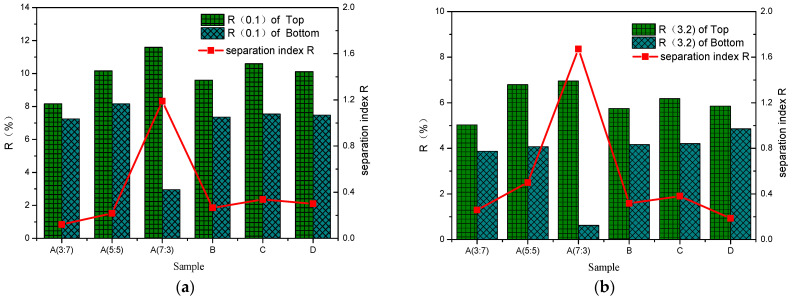
*R* based on different positions of the tube and separation index *R* for SBR/PP composite-modified asphalt. (**a**) Stress levels of 0.1 kPa. (**b**) Stress levels of 3.2 kPa.

Figure 3 shows the change in the elastic recovery rate and separation index *R* of different modified asphalts. Under the action of low stress, with the increase in the rubber-to-plastic ratio, the flexible recovery rate of the top part of the separation tube gradually increases. The addition of compatibilizer can cause the elastic recovery rate of SBR/PP composite-modified asphalt to be reduced. At the same time, the use of crosslinking agents has little effect on the elastic recovery rate of SBR/PP composite-modified asphalt. When a high-stress load is applied, the elastic recovery rate of the bottom sample of sample A (7:3) decreases, and its separation index *R* increases significantly, indicating that the thermoplastic elastomer in the SBR/PP composite-modified asphalt floats and aggregates. This makes the content of thermoplastic elastomer at the bottom of the tube relatively low, which causes large deformation under the action of the high-stress load. The deformation amount should not be recovered. This shows that the recovery rate *R* index of modified asphalt mainly reflects the elastic recovery ability of SBR in SBR/PP composite-modified asphalt. Under the action of low stress, the higher the proportion of SBR, the stronger its elastic recovery ability. Under the action of high strain, due to the action of multiple alternating loads, SBR produces displacement damage and ring breakage, which makes it lose part of its elastic recovery ability.

**Figure 4:**
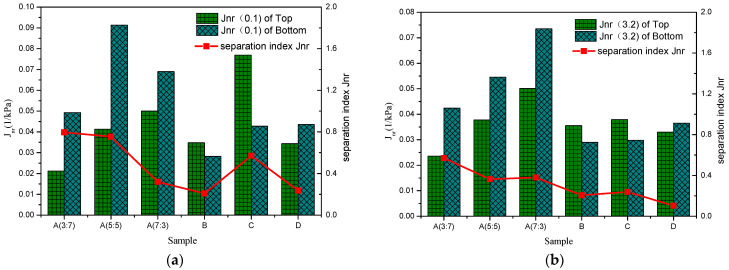
*J_nr_* based on different positions of the tube and separation index *J_nr_* for SBR/PP composite-modified asphalt. (**a**) Stress levels of 0.1 kPa. (**b**) Stress levels of 3.2 kPa.

The non-recoverable creep compliance *J_nr_* reflects the cumulative deformation capacity of the modified asphalt; the smaller the value, the lower the cumulative deformation and the stronger the deformation resistance. It can be seen from Figure 4 that, with the increase in the rubber-to-plastic ratio, the deformation resistance of the modified asphalt gradually decreases under the action of high and low stress, which is mainly due to the low PP content. This makes the instantaneous strain relatively large, resulting in the irrecoverable strain being relatively high. It shows that the non-recoverable creep compliance *J_nr_* mainly reflects the plastic deformation resistance of the PP polymer in the modified asphalt.

From the comparison between Figure 3 and Figure 4, it can be seen that, with the increase in the rubber-to-plastic ratio, the evaluation of separation index *R* and separation index *J_nr_* show opposite trends. The main reason may be that the two evaluation indexes reflect the different internal characteristics of modified asphalt. The separation index *R* mainly shows the elastic recovery ability of SBR polymer in the modified asphalt. When the SBR content in the thermoplastic elastomer is high, more SBR floats and aggregates with the thermoplastic elastomer during high-temperature storage, resulting in a significant improvement in the elastic recovery ability of the modified asphalt in the top position of the separator, and increasing the value of the separation index *R*. For the separation index *J_nr_*, this mainly reflects the plastic deformation resistance of the PP polymer in the modified asphalt. When the PP content in the thermoplastic elastomer is low, only a small amount of PP floats and aggregates with the thermoplastic elastomer, and its contribution to deformation resistance is negligible, which makes the distinction of the separation index *J_nr_* not obvious. On the other hand, the ageing of asphalt will further reduce the plastic deformation capacity of the modified asphalt on the top and bottom parts of the separator, making the non-recoverable creep compliance *J_nr_* of the modified asphalt on the top and bottom of the separator relatively small, resulting in a reduction in the value of the evaluation index separation index *J_nr_*.

To sum up, the evaluation index separation index *R* mainly reflects the stability of polymer SBR in composite-modified asphalt, and the separation index *J_nr_* mainly reflects the stability of polymer PP in composite-modified asphalt. Therefore, in view of the differences reflected by the two evaluation methods, it is necessary to normalize based on the proportion of composite polymers and further propose a comprehensive separation index evaluation index [37]:

(3) $$separation\;index=\frac{\sum_{i=1}^{3}\frac{{SIR}_{0.1i}{+SIR}_{3.2i}}{P_{SBRi}}+\sum_{j=1}^{3}\frac{{SIJ}_{nr0.1j}+{SIJ}_{nr3.2j}}{P_{PPj}}}{2}$$

*SIR*_0.1_ represents the separation index *R* under the condition that the applied load is 0.1 kPa;

*SIR*_3.2_ represents the separation index *R* under the condition that the applied load is 3.2 kPa;

*SIJ_nr_*_0.1_ represents the separation index *J_nr_* under the condition that the applied load is 0.1 kPa;

*SIJ_nr_*_3.2_ represents the separation index *J_nr_* under the condition that the applied load is 3.2 kPa;

*P_SBR_* represents the proportion of SBR in SBR/PP thermoplastic elastomer. When *i* = 1, 2, 3, *P_SBRi_* is 0.3, 0.5, 0.7, respectively; *SIR*_0.1*i*_ and *SIR*_3.2*i*_ are the corresponding separation index *R* values, respectively;

*P_PP_* represents the proportion of *PP* in SBR/PP thermoplastic elastomer. When *j* = 1, 2, 3, *P_PPi_* is 0.3, 0.5, 0.7, respectively; *SIJ_nr_*_0.1*j*_ and *SIJ_nr_*_3.2*j*_ are the corresponding separation index *J_nr_* values, respectively.

**Figure 5:**
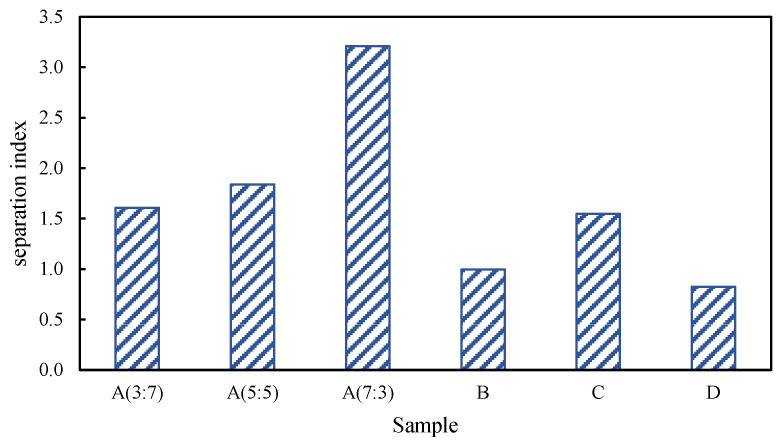
Separation index for SBR/PP composite-modified asphalt.

Figure 5 shows the separation index of different types of modified asphalt. With the increase in the rubber-to-plastic ratio, the high-temperature storage stability of composite-modified asphalt gradually decreases; while the difference between the separation index values of sample A (3:7) and A (5:5) is slight, the change range of sample A (7:3) is significantly increased, and the separation index value of sample A (3:7) increases by 100%. With the addition of a compatibilizer, the separation index of composite-modified asphalt decreased by 44%, indicating that its storage stability was improved. In contrast, the addition of a crosslinker had little effect on the storage stability of composite-modified asphalt. There was no significant difference in the separation index value compared with sample A (5:5). In conclusion, the addition of a compatibilizer can significantly reduce the separation index of SBR/PP-modified asphalt, indicating that the probability of separation of thermoplastic elastomer is reduced, which can further improve the thermal stability of SBR/PP composite-modified asphalt.

### 3.4. Analysis of Compatibility Mechanism of SBR/PP Composite-Modified Asphalt

#### 3.4.1. Frequency Sweep

**Figure 6:**
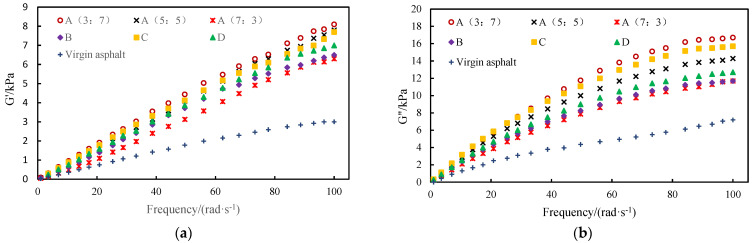
Frequency scanning curve of SBR/PP composite-modified asphalt. (**a**) Elastic modulus; (**b**) Viscous modulus.

Based on the frequency scanning test, the elastic modulus *G*′ and viscous modulus *G*″ of SBR/PP composite-modified asphalt were analyzed. The test results are shown in Figure 2. It can be seen from Figure 6 that, with the increase of load frequency, the elastic modulus and viscous modulus of SBR/PP composite-modified asphalt gradually increase, indicating that its high-temperature deformation resistance will be improved under high-frequency load. This phenomenon brings to mind examples from actual traffic scenarios, such as asphalt pavements near road intersections and bus stops, as these are more prone to rutting or deformation in China. The main reason for this is the low speed of vehicles, the long duration of driving loads, and the increased contact time and area between vehicles’ tires and the asphalt pavement, which leads to greater deformation of the pavement material.

In other words, as the frequency of traffic loading (or driving speed) increases, the modulus of elasticity and viscous modulus of the pavement material gradually increases. On the contrary, the lower the loading frequency, the smaller the elastic modulus and viscous modulus of the pavement material. Therefore, the deformation resistance of pavement materials is relatively weak at low loading frequencies. On the contrary, when traffic load is applied to asphalt pavement, the longer the load duration, the larger the cumulative deformation, the smaller the elastic recovery, and the lower the deformation resistance. Therefore, we think that the relationship curve between elastic modulus and loading frequency in Figure 6 can theoretically explain this phenomenon.

Under the same load frequency, compared with the base asphalt, the elastic modulus and viscous modulus of the SBR/PP composite-modified asphalt are greatly improved, and the high-temperature deformation resistance is improved. With the increase in the rubber-to-plastic ratio *M*_SBR_:*M*_PP_, the elastic modulus and viscous modulus of SBR/PP composite-modified asphalt gradually decreased, indicating that its high-temperature deformation resistance was weakened. This is mainly due to the relatively strong mechanical properties of the polypropylene crosslinked structure; the lower its proportion in the SBR/PP thermoplastic elastomer, the poorer the high-temperature deformation resistance of the modified asphalt. In hot areas in summer, an SBR/PP thermoplastic elastomer with a rubber-to-plastic ratio (*M*_SBR_:*M*_PP_) of 3:7 may be preferred to change the asphalt. When the compatibilizer was added, the elastic modulus and viscous modulus of SBR/PP composite-modified asphalt were further reduced. When a crosslinking agent was added, the elastic modulus and viscous modulus of SBR/PP composite-modified asphalt were increased, and the high-temperature deformation resistance was improved.

#### 3.4.2. Cole–Cole Diagram Analysis

The Cole–Cole diagram is an important method to study the rheological behavior of blended polymers. It represents dynamic viscosity *η*″ (*η*″ = *G*′/*ω*) and loss viscosity *η*′ (*η*′ = *G″*/*ω*), allowing the relationship between the rheological properties of different polymer composite-modified structures to be studied. Different curve forms represent a mechanism of relaxation. The relaxation phenomenon in the high-frequency region is mainly due to the relaxation of the continuous phase, and in the low-frequency region it is primarily due to the variability of the dispersed phase. This reveals information on relaxation mechanism and blending uniformity [38,39]. Therefore, in order to better evaluate the compatibility of SBR/PP thermoplastic elastomer and asphalt, the test analyzes the compatibility characteristics of modified asphalt through the study of Cole–Cole diagram types, and the Cole–Cole diagram of general homogeneous system shows a semicircular arc curve. The Cole–Cole diagram of the two-phase system shows an incomplete semicircular arc curve.

**Figure 7:**
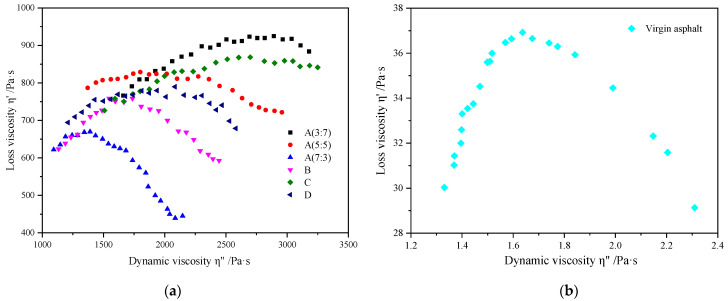
Cole–Cole diagram analysis of SBR/PP composite-modified asphalt. (**a**) Sample A, Sample B, Sample C and Sample D. (**b**) Virgin asphalt.

Figure 7a,b shows the Cole–Cole diagrams of different modified asphalts. The Cole–Cole diagrams of the matrix asphalt are closest to the semicircular arc curve, mainly because the base asphalt belongs to a homogeneous system, and there is no significant difference in the relaxation phenomenon between the high- and low-frequency ranges. With the increase in the rubber-to-plastic ratio, *M*_SBR_:*M*_PP_, the radian of its Cole–Cole diagram gradually decreases. When the rubber-to-plastic ratio, *M_SBR_*:*M_PP_*, is 3:7, the SBR/PP composite-modified asphalt tends to form a semicircle, indicating that the system compatibility of the middle two-phase structure is relatively good. When the rubber-to-plastic ratio, *M_SBR_*:*M_PP_*, is 5:5, a “small tail” appears at the end of the arc shape, which shows the sign of deviation from the semicircle. When the ratio of rubber to plastic is 7:3, the curved tail shows an obvious hook shape, indicating that under the condition of this rubber plastic ratio, the composite-modified asphalt shows different behavior from the homogeneous system, which is due to SBR/PP thermoplastic elastomer having other interface effects or non-uniform dispersion in the matrix asphalt, which makes the composite-modified asphalt show two relaxation mechanism phenomena. The above analysis shows that the compatibility of SBR/PP thermoplastic elastomer with asphalt is relatively poor. With the increase in the rubber-to-plastic ratio, the tendency of SBR/PP thermoplastic elastomer to swell and disperse in asphalt is gradually weakened, which is also consistent with the change rule of the high-temperature (mentioned above) storage stability of modified asphalt.

When the compatibilizer is added, it can be seen from the B-arc in the Cole–Cole diagram that the curve basically presents a semicircle shape, indicating that the compatibilizer can promote the dispersion of the SBR/PP thermoplastic elastomer in asphalt, making the compatibility of the system relatively the best. When the crosslinking agent is added, the C-arc in the Cole–Cole diagram basically does not show the shape of the arc, indicating that the addition of crosslinking agent is not conducive to the dispersion of SBR/PP thermoplastic elastomer in asphalt; When compatibilizers and crosslinkers are added at the same time, the D-arc in the Cole–Cole diagram can also show a better semicircular shape, and the blend system is relatively uniform.

#### 3.4.3. Microstructure Analysis of SBR/PP Composite-Modified Asphalt

**Figure 8:**
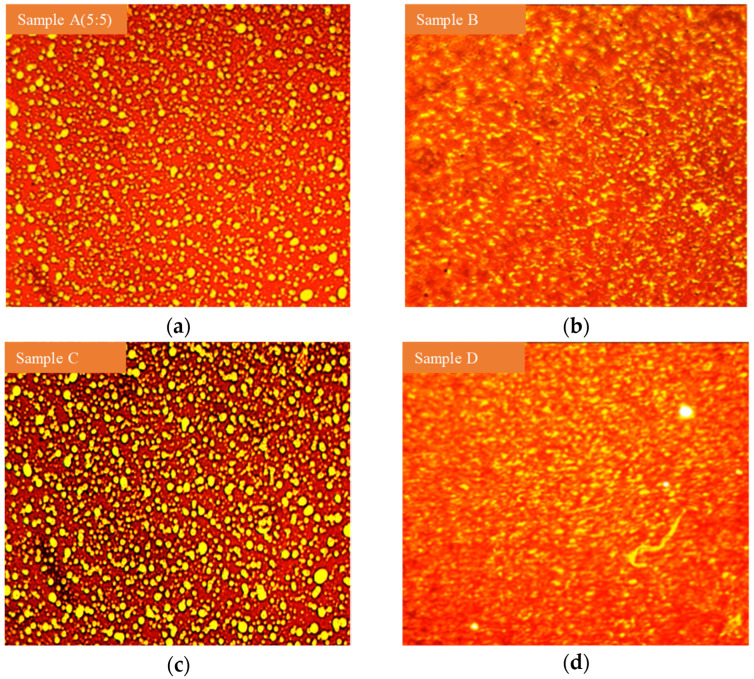
Fluorescence micrograph of SBR/PP composite-modified asphalt. (**a**) Sample A (5:5). (**b**) Sample B. (**c**) Sample C. (**d**) Sample D.

The SBR/PP thermoplastic elastomer exhibited fluorescence when illuminated by red light, while the asphalt showed no fluorescence reflection. Therefore, the distribution of SBR/PP thermoplastic elastomer can be identified from the pitch phase. The yellow phase in the picture represents the SBR/PP thermoplastic elastomer, and the red or dark red phase represents the asphalt. For sample A (5:5), it can be seen from Figure 8 that the SBR/PP thermoplastic elastomer is uniformly distributed in the asphalt matrix in the form of island particles, and there are relatively many “agglomerated” particles in SBR/PP phase. After adding compatibilizer, it can be seen from sample B that the phase of SBR/PP thermoplastic elastomer swells and disperses more fully, and its particle size decreases, mainly because the compatibilizer in SBR/PP thermoplastic elastomer can reduce the surface tension of SBR/PP blend polymer, make SBR/PP thermoplastic elastomer more compatible with asphalt, reduce the occurrence of “agglomeration” of SBR/PP phase, and effectively reduce the size of the dispersed phase. Thus, the phase structure of modified asphalt changes. After adding a compatibilizer and crosslinking agent, it can be seen from sample D that the particle size of SBR/PP thermoplastic elastomer is small, and crosslinking occurs between particles, which promotes the formation of an interpenetrating network of SBR, PP and asphalt. Compared with sample B, the degree of crosslinking between particles is more significant, and the binding with asphalt is optimized.

**Figure 9:**
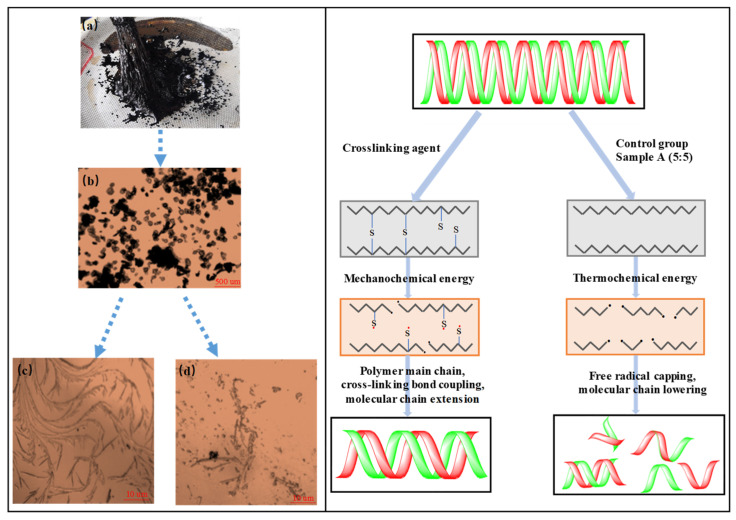
Action mechanism of SBR/PP thermoplastic elastomer modified asphalt. (**a**) SBR/PP residual polymer extracted from modified asphalt. (**b**) Microstructure image of SBR/PP-modified asphalt extracted polymer after mechanical stirring for 20 min. (**c**) SBR/PP polymer extracted from sample C. (**d**) Microstructure image of SBR/PP polymer extracted from sample B.

To visually demonstrate the impact of the crosslinking agent on the polymer, a comparative analysis was conducted to study the effects of adding and not adding the crosslinking agent. Initially, the thermally modified asphalt was passed through a sieve with a pore size of 1.18 mm to retain a significant amount of polymer residue on the sieve. Subsequently, the sieve was washed with kerosene three to five times, followed by sampling. The microscopic structure was then observed using a fluorescence microscope. As shown in Figure 9, the macromolecular chains of SBR/PP with long-chain structures will be broken by strong mechanical shearing force and thermochemical energy during the preparation process. In Figure 9c, the presence of the crosslinking agent leads to a polymer phase structure that tends more towards continuous crosslinking. This phenomenon aids in reinforcing the network structure of the SBR/PP thermoplastic elastomer, increasing the degree of particle crosslinking. During the asphalt swelling process, it facilitates the formation of a three-dimensional crosslinked network, thereby significantly improving its rheological performance and high-temperature stability.

To sum up, the addition of SBR/PP thermoplastic elastomer can change the phase transition structure of an asphalt system, under the condition of using a compatibilizer and a crosslinking agent. It can improve the continuity and compatibility of SBR/PP thermoplastic elastomer in asphalt, which is also consistent with the Cole–Cole diagram analysis of modified asphalt. The reasons for the improvement of high-temperature storage stability and rheological properties of modified asphalt were further explained after SBR/PP thermoplastic elastomer and asphalt were crosslinked to form a network structure. At the same time, the three-dimensional network structure plays a reinforcing role in the whole system, so that the modified asphalt has better anti-cracking ability at low temperatures and anti-deformation ability at high temperatures, which reflects the compound effect of styrene-butadiene rubber and polypropylene. In future studies, environmental ageing phenomena and the result of stimulated weathering conditions tests and wheel track tests will validate the long-term performance of modified asphalt pavements.

## 4. Conclusions

In this study, the performance optimization effect is based on the principle of melt blending. Firstly, SBR/PP thermoplastic elastomer particles were prepared using plasticizing technology to study the influence of different rubber plastic ratios on the thermal stability of SBR/PP thermoplastic elastomer-modified asphalt. Through the multi-stress creep recovery test, the evaluation method of the thermal stability of modified asphalt was designed, and the comprehensive separation index evaluation index was proposed. Finally, the compatibility between SBR/PP thermoplastic elastomer and asphalt was studied using the Cole–Cole diagram and microstructure image, and its modification mechanism was analyzed in detail. According to the test results in this study, the following conclusions can be drawn.

The SBR/PP thermoplastic elastomer particles prepared by plastic refining technology can improve the rheological properties of asphalt. The ratio design tests show that different rubber-to-plastic ratios have significant effects on the thermal stability of modified asphalt. The high-temperature thermal stability gradually decreases with the increase of the proportion of SBR in the composite polymer, up to 100%. After the use of additives, it can promote the homogeneous dispersion and good network structure of SBR/PP thermoplastic elastomer in the asphalt system, enhance the compatibility of the three-phase structure, and improve the high-temperature storage stability of SBR/PP composite-modified asphalt.

Based on the multi-stress creep recovery test, the design evaluation index, separation index *R*, mainly reflects the stability of polymer SBR in the composite-modified asphalt. In contrast, the separation index *J_nr_* mainly reflects the stability of polymer PP in the composite-modified asphalt. Through the normalization of the composite polymer percentage, and then putting forward a comprehensive thermal stability evaluation index separation index, the evaluation index—by optimizing the influence of different polymers on asphalt performance weights to equalize their contribution—can more reasonably reflect the overall changes in the composite-modified asphalt high-temperature storage stability law. This allows composite-modified asphalt thermal stability evaluation to provide scientific guidance.

The Cole–Cole plot analysis shows that the compatibility of modified asphalt has some correlation with the degree of semicircular arc of the Cole–Cole plot. The more significant its semicircular arc curve, the better the compatibility and homogeneous dispersion effect of SBR/PP particles and asphalt. Its analysis conclusion is also consistent with microstructure analysis, which can be used as a reference evaluation index of thermal stability of composite-modified asphalt, and to strengthen the joint analysis between microstructure characterization information and mechanical property parameters, which helps to improve the quality control of SBR/PP composite-modified asphalt.

## Figures and Tables

**Table 1:** Technical indexes of SK 70 asphalt.

Test Index	Test Result	Specification Requirement	Test Method
Penetration (25 °C)/0.1 mm	69	60~80	T0604
Softening point/°C	49	≥45	T0606
Ductility (15 °C)/cm	>100	≥100	T0605

**Table 2:** Mix design of SBR/PP thermoplastic elastomer.

Sample Number	M SBR:M PP *	Compatibilizer/%	Crosslinker/%
A (3:7)	3:7	0	0
A (5:5)	5:5	0	0
A (7:3)	7:3	0	0
B	5:5	1.5	0
C	5:5	0	0.5
D	5:5	1.5	0.5

* The additive based on the mass of thermoplastic elastomer.

**Table 3:** Technical indexes of SBR/PP composite-modified asphalt.

Sample Number	Penetration (25 °C)/0.1 mm	Softening Point/°C	Ductility (5 °C)/cm	Softening Point Difference/°C
Virgin asphalt	69	49	6	/
A (3:7)	41	68	11	1.9
A (5:5)	48	64	59	2.1
A (7:3)	57	58	83	2.2
B	54	62	101	1.8
C	47	66	88	2.0
D	51	65	90	1.8

**Table 4:** Rheological parameters of PG grading of SBR/PP composite-modified asphalt.

Sample Number	Dynamic Shear Rate 10 Rad/s (1.59 Hz)					Bending Creep (BBR, 60 s)			PG
	Unaged		RTFOT Ageing			PAV Ageing			
	Temperature/°C	G*/sin *δ* /kPa	G*/sin *δ* /kPa	Temperature/°C	G*·sin *δ* /Mpa	Temperature/°C	Stiffness Modulus/Mpa	*m*	
Virgin asphalt	64	1.6	2.3	25	4.4	−12	57.21	0.38	PG 64-22
A (3:7)	82	1.8	2.7	37	2.7	−6	214.59	0.37	PG 82-16
A (5:5)	82	1.4	2.5	34	2.0	−12	200.28	0.33	PG 82-22
A (7:3)	76	1.7	3.3	31	1.9	−12	174.53	0.39	PG 76-22
B	76	1.6	3.5	28	3.6	−18	39.23	0.51	PG 76-28
C	82	1.7	2.5	34	1.8	−12	186.54	0.43	PG 82-22
D	82	1.5	2.4	31	1.7	−18	68.84	0.45	PG 82-28

## Data Availability

Data are available on request to the authors.

## References

[1] Yan K., You L., Wang D. (2019). High-Temperature Performance of Polymer-Modified Asphalt Mixes: Preliminary Evaluation of the Usefulness of Standard Technical Index in Polymer-Modified Asphalt. Polymers.

[2] Habbouche J., Hajj E.Y., Sebaaly P.E., Piratheepan M. (2020). A Critical Review of High Polymer-Modified Asphalt Binders and Mixtures. Int. J. Pavement Eng..

[3] Xia T., Zhang A., Xu J., Chen X., Xia X., Zhu H., Li Y. (2021). Rheological Behavior of Bitumen Modified by Reclaimed Polyethylene and Polypropylene from Different Recycling Sources. J. Appl. Polym. Sci..

[4] Zhang H., Gong M., Gao D., Yang T., Huang Y. (2020). Comparative Analysis of Mechanical Behavior of Composite Modified Asphalt Mixture Based on PG Technology. Constr. Build. Mater..

[5] Ameli A., Norouzi N., Khabbaz E.H., Babagoli R. (2020). Influence of Anti Stripping Agents on Performance of Binders and Asphalt Mixtures Containing Crumb Rubber and Styrene-Butadiene-Rubber. Constr. Build. Mater..

[6] Ming L.Y., Feng C.P., Siddig E.A.A. (2018). Effect of Phenolic Resin on the Performance of the Styrene-Butadiene Rubber Modified Asphalt. Constr. Build. Mater..

[7] Wu S., Montalvo L. (2021). Repurposing Waste Plastics into Cleaner Asphalt Pavement Materials: A Critical Literature Review. J. Clean. Prod..

[8] Audy R., Enfrin M., Boom Y.J., Giustozzi F. (2022). Selection of Recycled Waste Plastic for Incorporation in Sustainable Asphalt Pavements: A Novel Multi-Criteria Screening Tool Based on 31 Sources of Plastic. Sci. Total Environ..

[9] Duggal P., Shisodia A.S., Havelia S., Jolly K., Adhikari S., Bhattacharjee B., Bhattacharjee J. (2020). Use of Waste Plastic in Wearing Course of Flexible Pavement. Proceedings of the Advances in Structural Engineering and Rehabilitation.

[10] Jerin T., Jahan N., Jaya J.D., Sami N.A., Hossain M.I., Islam M.R. Polypropylene Modified Asphalt Mix to Improve Wearing Course of Flexible Pavement. Proceedings of the International Conference on Transportation and Development 2020: Highway and Airfield Pavements.

[11] Cheng P., Li Y., Zhang Z. (2020). Effect of Phenolic Resin on the Rheological and Morphological Characteristics of Styrene-Butadiene Rubber-Modified Asphalt. Materials.

[12] Vamegh M., Ameri M., Chavoshian Naeni S.F. (2019). Performance Evaluation of Fatigue Resistance of Asphalt Mixtures Modified by SBR/PP Polymer Blends and SBS. Constr. Build. Mater..

[13] Wang H., Du Z., Liu G., Luo X., Yang C. (2023). Effect of Long-Term Aging on Fatigue and Thermal Cracking Performance of Polyphosphoric Acid and Styrene–Butadiene–Styrene-Modified Bio-Blend Bitumen. Polymers.

[14] Tam H.L.S., Tang D.T.W., Leung W.Y., Ho K.M., Greenfield P.F. (2004). Performance Evaluation of Hybrid and Conventional Sequencing Batch Reactor and Continuous Processes. Water Sci. Technol..

[15] Ren Z., Zhu Y., Wu Q., Zhu M., Guo F., Yu H., Yu J. (2020). Enhanced Storage Stability of Different Polymer Modified Asphalt Binders through Nano-Montmorillonite Modification. Nanomaterials.

[16] Li J., Jia S., Yang D., Li X., Li D., Ye X., Guan P., Zhao Y. (2022). Reutilization of Crystalline Waste Plastics for Modified Asphalt Using Twin-Screw Extrusion: Polyethylene and Polypropylene as Typical Subjects. J. Appl. Polym. Sci..

[17] Rohayzi N.F., Katman H.Y.B., Ibrahim M.R., Norhisham S., Rahman N.A. (2023). Potential Additives in Natural Rubber-Modified Bitumen: A Review. Polymers.

[18] Liu G., Fang S., Wang Y., Liu J., Liang Y., Cao T., Liu Q. (2023). Emission of Volatile Organic Compounds in Crumb Rubber Modified Bitumen and Its Inhibition by Using Montmorillonite Nanoclay. Polymers.

[19] Wang Z., Zeng D. (2021). Preparation of Devulcanized Ground Tire Rubber with Supercritical Carbon Dioxide Jet Pulverization. Mater. Lett..

[20] (1981). Polypropylene—Method of Determination of Normal Indices.

[21] Schaur A., Unterberger S.H., Lackner R. (2021). Impact of Molecular Structure of PP on Thermo-Rheological Properties of Polymer-Modified Bitumen. Constr. Build. Mater..

[22] Singh G., Kaur S., Kothari A.V., Naik D.G., Vyas P.B., Gupta V.K. (2009). Studies on the Influence of Molecular Weight and Isotacticity of Polypropylene on the Formation of Mesomorphic Phase. J. Appl. Polym. Sci..

[23] Luo J., Yang Y., Huang W., Xie C., Chen J., Liu H., Ren T., Huang X. (2022). Physical, Rheological, And Microsurface Characteristics of High-Viscosity Binder Modified with WMA Agents. Adv. Mater. Sci. Eng..

[24] Liu S., Cao W., Fang J., Shang S. (2009). Variance Analysis and Performance Evaluation of Different Crumb Rubber Modified (CRM) Asphalt. Constr. Build. Mater..

[25] Singh D., Girimath S. (2016). Investigation of Rheological Properties and Superpave PG of PMB Mixed with Reclaimed Asphalt Pavement Binders. Constr. Build. Mater..

[26] Jafari M., Babazadeh A., Aflaki S. (2015). Effects of Stress Levels on Creep and Recovery Behavior of Modified Asphalt Binders with the Same Continuous Performance Grades. Transp. Res. Rec..

[27] (2011). Test Specification for Asphalt and Asphalt Mixture in Highway Engineering.

[28] Lu X., Isacsson U., Ekblad J. (1999). Phase Separation of SBS Polymer Modified Bitumens. J. Mater. Civ. Eng..

[29] Gao L., Cai N., Fu X., He R., Zhang H., Zhou J., Kuang D., Liu S. (2021). Influence of PPA on the Short-Term Antiaging Performance of Asphalt. Adv. Civ. Eng..

[30] Ghuzlan K.A., Al-Khateeb G.G. (2013). Selection and Verification of Performance Grading for Asphalt Binders Produced in Jordan. Int. J. Pavement Eng..

[31] Xing C., Li M., Liu L., Yang R. (2023). Long-Term Aging Behavior of Plastic/Styrene Butadiene Rubber (SBR) Composite Modified Bitumen. Materials.

[32] Jahromi S.G., Ahmadi N.A., Vossugh S., Mortazavi M. (2012). Effects of Nanoclay on Rutting and Fatigue Resistance of Bitumen Binder. Int. J. Mater. Res..

[33] Ma X., Ni F., Chen R., Wang Y. (2010). Evaluation of Low-Temperature Performance of Porous Asphalt Mixture. J. Highw. Transp. Res. Dev..

[34] Yan K., Li Y., Long Z., You L., Wang M., Zhang M., Diab A. (2022). Mechanical Behaviors of Asphalt Mixtures Modified with European Rock Bitumen and Waste Cooking Oil. Constr. Build. Mater..

[35] Vamegh M., Ameri M., Chavoshian Naeni S.F. (2020). Experimental Investigation of Effect of PP/SBR Polymer Blends on the Moisture Resistance and Rutting Performance of Asphalt Mixtures. Constr. Build. Mater..

[36] Pandey A., Islam S.S., Ransingchung R.N. G.D., Ravindranath S.S. (2023). Comparing the Performance of SBS and Thermoplastics Modified Asphalt Binders and Asphalt Mixes. Road Mater. Pavement Des..

[37] Yue S., Wang J.-S., Wu T., Wang H. (2010). A New Separation Measure for Improving the Effectiveness of Validity Indices. Inf. Sci..

[38] Liang M., Xin X., Fan W., Ren S., Shi J., Luo H. (2017). Thermo-Stability and Aging Performance of Modified Asphalt with Crumb Rubber Activated by Microwave and TOR. Mater. Des..

[39] Rolere S., Cartault M., Sainte-Beuve J., Bonfils F. (2017). A Rheological Method Exploiting Cole-Cole Plot Allows Gel Quantification in Natural Rubber. Polym. Test..

